# Characterization of Zebrafish von Willebrand Factor Reveals Conservation of Domain Structure, Multimerization, and Intracellular Storage

**DOI:** 10.1155/2012/214209

**Published:** 2012-09-24

**Authors:** Arunima Ghosh, Andy Vo, Beverly K. Twiss, Colin A. Kretz, Mary A. Jozwiak, Robert R. Montgomery, Jordan A. Shavit

**Affiliations:** ^1^Life Sciences Institute, University of Michigan, Ann Arbor, MI 48109, USA; ^2^Department of Pediatrics, University of Michigan, Room 8301 Medical Science Research Building III, 1150 W. Medical Center Drive, Ann Arbor, MI 48109-5646, USA; ^3^Blood Research Institute, Medical College of Wisconsin, Milwaukee, WI 53226, USA

## Abstract

von Willebrand disease (VWD) is the most common inherited human bleeding disorder and is caused by quantitative or qualitative defects in von Willebrand factor (VWF). VWF is a secreted glycoprotein that circulates as large multimers. While reduced VWF is associated with bleeding, elevations in overall level or multimer size are implicated in thrombosis. The zebrafish is a powerful genetic model in which the hemostatic system is well conserved with mammals. The ability of this organism to generate thousands of offspring and its optical transparency make it unique and complementary to mammalian models of hemostasis. Previously, partial clones of zebrafish *vwf* have been identified, and some functional conservation has been demonstrated. In this paper we clone the complete zebrafish *vwf* cDNA and show that there is conservation of domain structure. Recombinant zebrafish Vwf forms large multimers and pseudo-Weibel-Palade bodies (WPBs) in cell culture. Larval expression is in the pharyngeal arches, yolk sac, and intestinal epithelium. These results provide a foundation for continued study of zebrafish Vwf that may further our understanding of the mechanisms of VWD.

## 1. Introduction

 Vertebrates possess a complex closed circulatory system that requires balanced coordination of various factors that serve to maintain blood flow as well as prevent exsanguination when the system is breached. This is known as hemostasis and consists of a complex array of cellular elements, as well as a network of proteins known as the coagulation cascade. The latter have been highly conserved at the genomic level throughout vertebrate evolution, including mammals, birds, reptiles, and fish [1–3].

One of the central components of coagulation is von Willebrand factor (VWF), deficiencies of which are the basis for the bleeding disorder von Willebrand disease (VWD). The mammalian *VWF *gene consists of 52 exons, and the largest, exon 28, contains several functional domains that are frequently mutated in VWD [4]. VWF is a 260 kDa (kilodalton) secreted glycoprotein that assembles into multimers of over 10,000 kDa [5]. At sites of injury, high molecular weight VWF multimers bind to receptors in the vascular subendothelium and tether platelets to form the primary hemostatic plug [6]. Much of our knowledge of VWF function is derived from characterization of mutations in humans and various mammalian model organisms, including mouse, dog, horse, cat, pig, and rabbit [7, 8]. However, relatively little information is available in other vertebrate models, such as the teleost *Danio rerio* (zebrafish). Teleost fish possess highly conserved orthologs of nearly all blood coagulation factors [1, 3] and have been shown to develop thrombosis in response to a laser-induced injury [9]. Zebrafish embryonic development is external, rapid, and transparent, greatly simplifying phenotypic screening. Circulation begins approximately 24 hours after fertilization, and vascular development has been well characterized [10]. Forward genetic screens with chemical mutagenesis have been performed to study cardiogenesis, vasculogenesis, and angiogenesis [11–14].

Recently exon 28 was cloned from zebrafish, and conservation of several VWF functions was demonstrated [15], and *in silico *assembly of full length zebrafish  *vwf* has also been described [16]. We now report cloning and characterization of the full length zebrafish *vwf* cDNA. Zebrafish Vwf demonstrates conservation of primary human VWF domain structure, as well as the ability to form pseudo-Weibel-Palade bodies (WPBs) and large multimers in cell culture. Unlike mammalian species, at the stages examined it does not appear to be expressed widely in developing endothelium.

## 2. Material and Methods

### 2.1. Cloning of Full Length Zebrafish *vwf* cDNA

Total mRNA was prepared from a single adult zebrafish using TRIzol (Invitrogen, Carlsbad, California). Total cDNA was synthesized with Superscript III reverse transcriptase after priming with random hexamers (Invitrogen). The *vwf* cDNA was assembled in four overlapping PCR amplified fragments using genomic sequence from Zv6 as a template to design primers (Table 1). Unique restriction sites contained in the overlapping sequences were used to sequentially assemble each of the four PCR products into the vector pCR4-TOPO (Invitrogen). The 5′ and 3′ UTRs (untranslated regions) were amplified by RACE (rapid amplification of cDNA ends, Ambion) with ends that overlapped unique restriction sites in the assembled clone. The external RACE primers were designed with restriction sites for the unique 5′ and 3′ vector sites, NotI and SpeI, respectively.

### 2.2. Multispecies Alignments

 Non-zebrafish VWF amino acid sequences were downloaded from the UCSC Genome Browser, http://genome.ucsc.edu/ [17], aligned using ClustalW2, http://www.ebi.ac.uk/Tools/msa/clustalw2/ [18, 19], with output display through BOXSHADE 3.21, http://www.ch.embnet.org/software/BOX_form.html. Domain comparisons were performed using two sequence protein BLAST (Basic Local Alignment Search Tool) with the default settings through the National Center for Biotechnology Information, http://blast.ncbi.nlm.nih.gov/.

### 2.3. Plasmid Cloning of *vwf *cDNA

The assembled *vwf *cDNA was cloned into pcDNA3.1/V5-HISA (Invitrogen), which has an 8 amino acid linker, producing pzVwf/V5-HISA. Since expression of tagged human VWF has been shown to be more robust with an 18–20 amino acid linker (R. Montgomery and S. Haberichter, unpublished observations), we amplified this linker from a human VWF/Myc-HIS construct (pVWF/Myc-HIS, linker sequence in Table 1) and cloned it into the 3′ XhoI-PmeI sites (derived from pcDNA3.1/V5-HISA) of pzVwf/V5-HISA, producing pzVwf/Myc-HIS. The human pVWF-EGFP plasmid contains the same linker sequence. p*fli*-zVwf-EGFP was constructed by inserting the *vwf *cDNA into Tol2-*fli-*EGFP [20] in frame with *egfp*.

### 2.4. Immunofluorescence Analysis

 HEK293T cells were maintained in DMEM (Sigma; St Louis, MO) supplemented with 10% fetal bovine serum, 100 U/mL penicillin, and 100 *μ*g/mL streptomycin (Sigma). Cells were grown on cover slips until they reached 50–80% confluence, followed by transfection using FuGENE (Roche, Penzberg, Germany) as per manufacturer's instructions. The transfected cover slips were washed in phosphate buffered saline (PBS) and fixed in 10% formalin at room temperature for 25 minutes, followed by fixation/permeabilization at 4°C for 10 minutes in 100% ice cold methanol. After rehydration in PBS for 5 minutes, the cells were incubated with mouse anti-Myc (Santa Cruz Biotechnology, Santa Cruz, California) and rabbit anti-calnexin (Novus Biologicals, Littleton, Colorado) antibodies at dilutions of 1 : 100 and 1 : 500, respectively, at 4°C overnight. Cells were then washed three times in PBS (5 minutes each) and incubated with goat anti-mouse antibody coupled to Alexa Fluor 488 and goat anti-rabbit antibody coupled to Alexa Fluor 594, both at 1 : 200 dilutions for 60 minutes at room temperature. After an additional three washes in PBS, the cover slips were mounted with Prolong Antifade Gold (Invitrogen) and viewed on an inverted Olympus (Melville, New York) confocal microscope. Processing was completed with Olympus FluoView version 5.0.

### 2.5. Vwf Multimer Analysis

HEK293T (human embryonic kidney) cells were cultured and transfected with pzVwf/V5-HISA or an untagged full length human VWF expressing plasmid (pCineoVWF), as previously described [21]. Conditioned medium from pzVwf/V5-HISA transfected cells was purified over nickel columns per manufacturer's instructions (GE Healthcare Life Sciences, Uppsala, Sweden). Supernatants were analyzed by electrophoresis through a 0.8% (w/v) HGT(P) agarose (FMC Bioproducts, Rockland, Maine) stacking gel and a 1.5% (w/v) HGT(P) agarose running gel containing 0.1% sodium dodecyl sulfate for 16 hours at 40 volts using the Laemmli buffer system and western blotting as previously described [21]. Primary antibodies were a 1 : 5 mixture of anti-V5 antibody (Invitrogen) and anti-HIS antibody (AbD Serotec, Oxford, United Kingdom) or a mixture of monoclonal anti-human VWF antibodies Avw1, 5, and 15 [22].

### 2.6. Maintenance of Zebrafish Lines and Production of Embryos

Adult zebrafish (AB, TL, EK) were maintained and bred according to standard methods [23]. Embryos collected immediately after fertilization were maintained at 28.5°C and treated with 1-phenyl-2-thiourea (PTU) at 6–8 hpf (hours post fertilization) until fixation in order to prevent pigment formation. At specific time points, embryos were dechorionated or euthanized with tricaine, fixed using 4% paraformaldehyde in PBS overnight at 4°C, and stored at −20°C in methanol up to one month [24]. 

### 2.7. RNA Isolation and cDNA Synthesis for RT-PCR of Embryos and Larvae

Total RNA was extracted from at least three biological replicates per experimental condition using TRIzol RNA isolation reagent (Invitrogen) according to the manufacturer's instructions. RNA (1 *μ*g) was reverse-transcribed using random hexamers and SuperScript III reverse transcriptase (Invitrogen). First-strand cDNA aliquots from each sample served as templates in PCR reactions using primers for *vwf.*

### 2.8. *In Situ* Hybridization

*In situ* hybridization was performed essentially as described with a few modifications [24]. Full length *vwf* cDNA in pCR4-TOPO was linearized with NotI and SpeI (antisense and sense transcripts, respectively) and transcribed *in vitro* using T3 and T7 (Ambion, Austin, Texas), respectively, with digoxigenin labeled nucleotides followed by alkaline hydrolysis per manufacturer's instructions (Roche). Alternatively, 424 and 441 bp fragments were amplified from full length cDNA using primers with SP6 or T7 overhangs (Table 1) and transcribed *in vitro* with digoxigenin labeled nucleotides. Prior to hybridization, riboprobes were heated to 80°C for 3–5 minutes and chilled immediately on ice for at least 5 minutes. Stained embryos were photographed using a Leica MXFLIII stereofluorescent microscope with an Olympus DP-70 digital camera. Embedding was in JB-4 resin as described [25], followed by sectioning at 4–6 *μ*m using a Leica RM2265 ultramicrotome. Imaging of sections was with an Olympus BX-51 upright light microscope and Olympus DP-70 high-resolution digital camera.

## 3. Results

### 3.1. Cloning and Characterization of Zebrafish *vwf* cDNA

**Figure 1:**
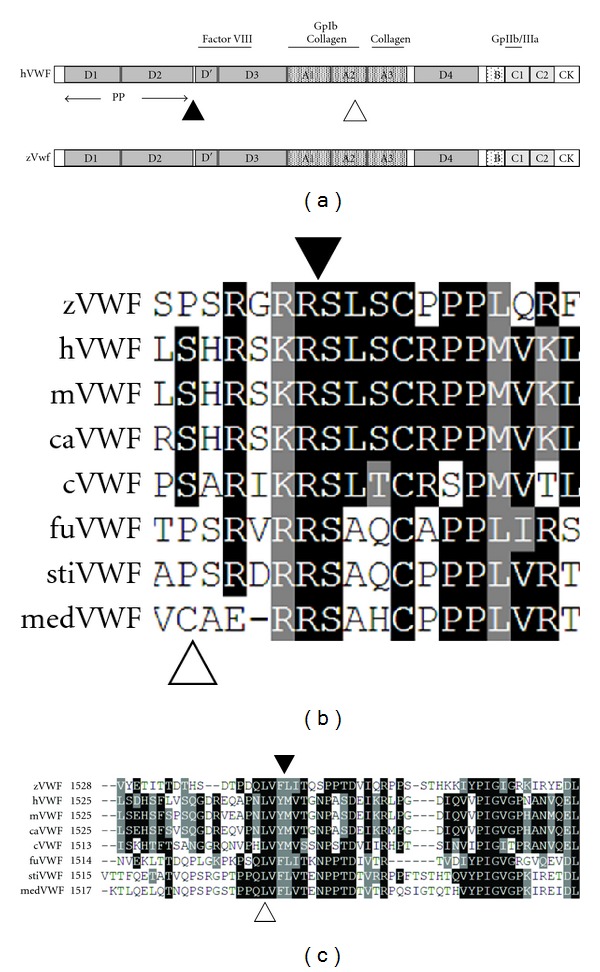
Domain organization of human VWF and multispecies alignment of the VWF propeptide and ADAMTS13 cleavage sites and flanking sequences. Sequence alignment was performed using ClustalW2 followed by output using BOXSHADE (Section 2). (a) Domain organization of human VWF. Upper notations indicate known protein-protein interaction domains (Gp: glycoprotein). The solid triangle indicates the propeptide (PP) cleavage site, and the open triangle indicates the ADAMTS13 cleavage site. “B” indicates domains B1–B3. (b) Alignment of sequences surrounding the Arg-Ser (RS, indicated by the solid triangle) human propeptide cleavage site demonstrates a high degree of conservation. Note the extended RX(R/K)R motif present in all species except for medaka. The open triangle indicates the presence of an unconserved cysteine in medaka Vwf. (c) Alignment at the human ADAMTS13 cleavage site (YM, indicated by the solid triangle) and flanking sequences demonstrates conservation of the Tyr-Met residues in mammalian and avian species, but a Phe-Leu putative site in teleost fish. The invariant Leu (human residue 1603) is indicated by a white triangle. z: zebrafish; h: human; m: mouse; ca: canine; c: chicken; fu: fugu; st: stickleback; med: medaka.

According to genomic sequence, the zebrafish *vwf* locus is located on chromosome 18 just downstream of *cd9*, maintaining conservation of synteny with mammalian species [15]. The full length *vwf *cDNA was assembled by RT-PCR of four overlapping fragments from total adult zebrafish cDNA, followed by RACE to complete the 5′ and 3′ UTRs (Section 2). The full length sequence is one amino acid shorter than human VWF with 46% overall identity (Table 2). Alignment of zebrafish Vwf to human VWF using BLAST shows clear delineation of all known domains (Figure 1(a)) with varying degrees of conservation (Table 2). The least conserved are the A1 and A2 domains, which encompass the entirety of exon 28 (Table 2). As in mammals, the *vwf *locus consists of 52 exons, but only spans 81 kb (kilobases), as opposed to 176 kb and 134 kb in the human and murine genomes, respectively. Previous iterations of the zebrafish genome (prior to Zv7) predicted that exon 28 was split into two exons [17]. Both sequence data from this report and previous work [15, 16] demonstrate clearly that the intervening sequence is actually exonic.

Other key features of human VWF are identifiable with varying degrees of conservation. The propeptide cleavage site, Arg-Ser, is highly conserved across all species examined except for medaka, and is a part of the extended RX(R/K)R motif (Figure 1(b)) [26]. The putative ADAMTS13 cleavage site in the A2 domain, Phe-Leu, is discernible due to mammalian orthology of flanking residues and is conserved across all fish species (Figure 1(c)). However, the presumed Phe-Leu cleavage site is only somewhat similar to the highly conserved mammalian and avian Tyr-Met cleavage sequence (Figure 1(c)). More importantly, there is conservation of a leucine orthologous to human Leu^1603^ (Figure 1(c)), which has been shown to be critical for ADAMTS13-mediated proteolysis of VWF [27].

A number of disulfide bonds are required for dimerization and multimerization of human VWF [6]. These are mediated by cysteines at positions 1099, 1142, and several in the C-terminal cystine knot (CK), at 2771, 2773, and 2811, all of which are conserved in zebrafish Vwf. In fact, nearly all cysteine residues are completely conserved, with the exception of Cys^1669^ and Cys^1670^, located at the C-terminus of the A2 domain [16] and absent in all fish species examined. There was one cysteine present solely in medaka, four residues N-terminal to the propeptide cleavage site, but its absence in other species makes its significance unclear. There is a cysteine in zebrafish Vwf at position 4, which is not conserved in mammalian species, although genomic sequence information for the other teleost species is absent in this region. 

### 3.2. Expression of Vwf in Mammalian Cell Culture

**Figure 2:**
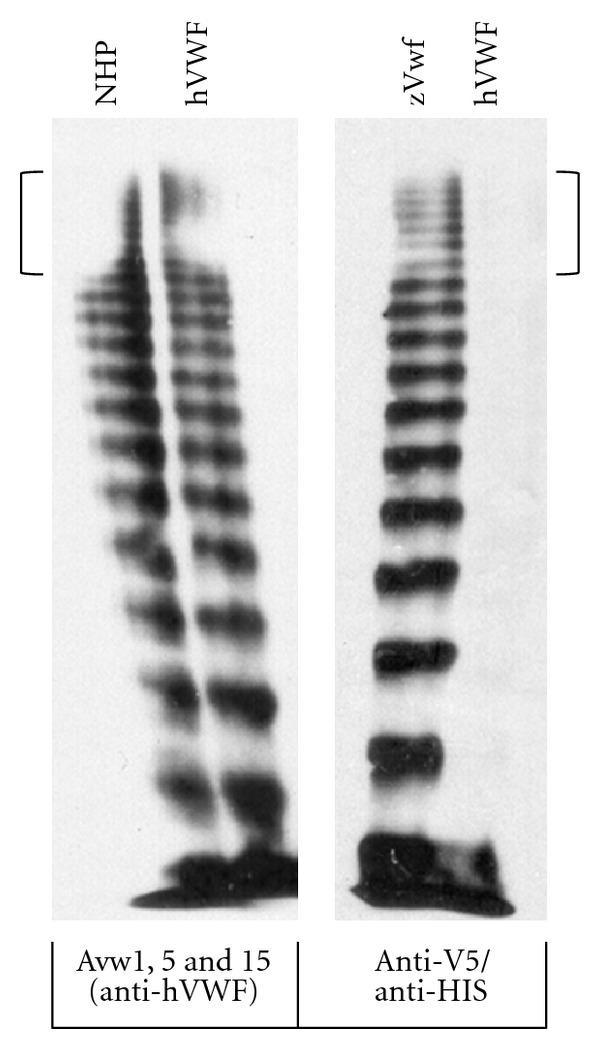
Multimerization of zebrafish Vwf in mammalian cell culture demonstrates high molecular weight multimers similar to human VWF. HEK293T cells were transfected with pzVwf/V5-HISA, expressing V5-HIS tagged zebrafish Vwf (zVwf), or pCineoVWF, expressing untagged human VWF (hVWF). Normal human plasma (NHP) and zebrafish and human supernatants were separated by agarose gel electrophoresis, transferred by western blotting, and detected with either a pool of monoclonal anti-hVWF antibodies (Avw1, 5, 15, left panel) or a mixture of anti-V5 and anti-HIS antibodies (for tagged zVwf, right panel). The anti-V5/HIS combination detects zVwf with a multimer pattern, including high molecular weight multimers, indistinguishable from that typically observed for human VWF (brackets indicate high molecular weight multimers for both zebrafish and human VWF).

In order to determine if zebrafish Vwf can multimerize, we expressed V5/HIS tagged *vwf* cDNA in HEK293T cells. A ladder of high molecular weight multimers was detected using a mixture of anti-V5 and anti-HIS antibodies (Figure 2). This included high molecular weight multimers similar in size to human VWF (Figure 2).

**Figure 3:**
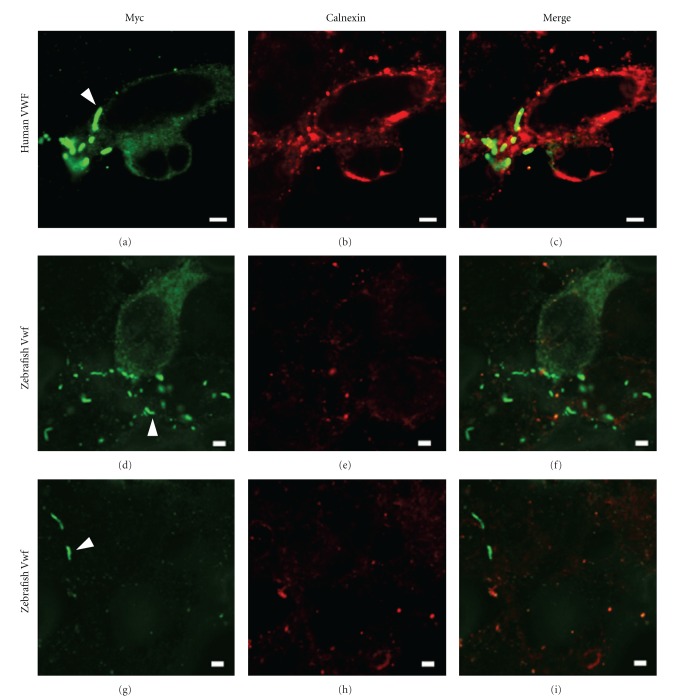
Zebrafish Vwf forms pseudo-Weibel-Palade bodies (pseudo-WPBs) in mammalian cell culture. pVWF/Myc-HIS (human VWF, (a–c)) or pzVwf/Myc-HIS (zebrafish Vwf, (d–i)) plasmids were transfected into HEK293T cells. Anti-Myc antibody conjugated to Alexa Fluor 488 (green channel, (a, d, g)) was used for detection and anti-calnexin antibody conjugated to Alexa Fluor 594 (red channel, (b, e, h)) labeled endoplasmic reticulum (ER). Both constructs demonstrate formation of elongated Myc positive and ER negative structures (absence of yellow signal in the merged panels, (c, f, i)) characteristic of pseudo-WPBs (examples are indicated in (a, d), and (g) by arrowheads). Scale bars, 2.5 *μ*m.

The zebrafish *vwf* cDNA was cloned into an expression vector in frame with a Myc-HIS tag using the same linker as a human *VWF* cDNA construct. The latter, when transfected into HEK293T cells, is known to form pseudo-WPBs [28, 29]. These structures are produced after VWF has been processed into high molecular weight multimers in the Golgi apparatus. Using an anti-Myc antibody we were able to identify elongated structures consistent with pseudo-WPBs in zebrafish *vwf *transfected cells (Figures 3(d) and 3(g)). These were morphologically similar to those found in human *VWF *transfected cells (Figure 3(a)). Staining with an anti-calnexin antibody to localize endoplasmic reticulum (ER, Figures 3(b), 3(e), and 3(h)) demonstrated no overlap between the structures (Figures 3(c), 3(f), and 3(i)), as expected for WPBs and pseudo-WPBs [28, 29].

### 3.3. Developmental Patterns of *vwf* Expression

**Figure 4:**
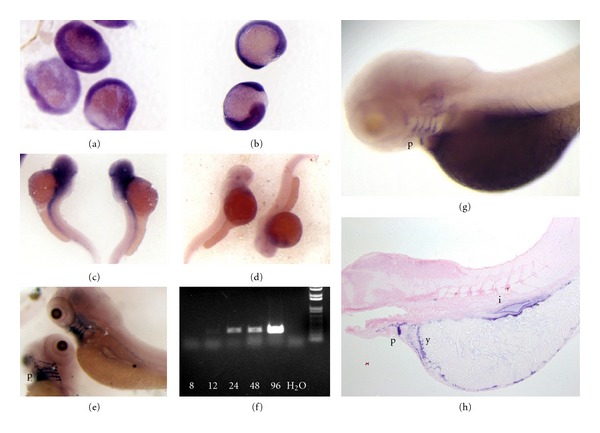
Developmental expression of *vwf* mRNA. Wild type zebrafish offspring were isolated from 8 to 120 hpf, fixed, and *in situ *hybridization was performed (Section 2). (a) Examination at 8 hpf demonstrates weak expression throughout the entire embryo, and staining was completely absent from a sense control. (b) Diffuse expression continues at 12 hpf (staining was completely absent from a sense control), followed by more restricted expression cranially with a stripe that extends caudally at 48 hpf (c). Figure 4(d) is a sense probe as negative control at 48 hpf. (e) 96 hpf shows strong expression in the pharyngeal arches. (f) RT-PCR of cDNA isolated from whole zebrafish embryos and larvae from 8–96 hpf. (g, h) Analysis at 120 hpf shows continued expression in the pharyngeal arches, as well as inner yolk sac layer and intestinal epithelium. Experiments in (a–e) used full length *vwf *riboprobes. Results in (g, h) are representative of hybridization with exon 28 and exon 47–52 riboprobes (Section 2, Table 1). Abbreviations: p: pharyngeal arches; y: inner layer of yolk sac; i: intestinal epithelium.

RT-PCR of whole embryos up to 96 hpf demonstrated increasing levels of *vwf *expression, with the most intense expression at 96 hpf (Figure 4(f)). Whole-mount *in situ* hybridization was used to localize expression from the middle of gastrulation (8 hpf) to 120 hpf. Expression of *vwf *is weakly detectable throughout the embryo at 8 hpf (Figure 4(a)). Stronger expression is observed in 12-hour embryos as a more diffuse pattern throughout the embryo (Figure 4(b)). At 48 hours there is diffuse expression cranially, which extends caudally (Figure 4(c)). At 96–120 hours, strong expression is present in the pharyngeal arches, intestinal epithelium, and inner layer of the yolk sac (Figures 4(e), 4(g), and 4(h)). 

## 4. Discussion

 VWD is due to quantitative or qualitative deficiency of VWF and has been described in several mammals, including human, horse, cat, pig, rabbit, and dog [7, 8]. Identification and characterization of the human *VWF *cDNA [30–33] enabled the eventual identification of many of these pathogenic mutations as well as partial or full length sequence information in numerous mammalian species [34]. The zebrafish genome project [35] assisted in the identification of much of the *vwf *cDNA [15, 16], but this did not include the complete 5′ and 3′ UTRS. We have now completed cloning and characterization of the full length zebrafish *vwf *cDNA.

We found that *vwf *displays widespread expression in early embryonic development and then becomes more restricted at the larval stage. Mammalian *VWF *is widely expressed in vascular endothelial cell beds of the adult mouse [36], and VWF protein is an established clinical pathologic marker of human vasculature [37]. However, it has not been examined in the developing vertebrate. We hypothesized that there would be widespread expression of zebrafish *vwf* in developing vasculature, but instead found an early broad and then later restricted pattern. A previous study in zebrafish identified Vwf protein expression within the vasculature at the larval stage, although the source was not determined [15]. Therefore one possible explanation for the discrepancy with our results is that larval intravascular Vwf is not produced in endothelial cells but rather comes from the yolk sac or pharyngeal arches. Alternatively, endothelial *vwf *mRNA expression might not be present until later in development. 

The expression seen in early embryonic development may possibly reflect maternally derived transcripts [38], while later expression is clearly of embryonic/larval origin. There is no prior evidence for a role of VWF in gastrulation, although the expression in the pharyngeal arches is intriguing. These structures develop into gills [39], the organs responsible for oxygen exchange in fish. The highest levels of mammalian *Vwf* mRNA expression have been identified in the lung [36], suggesting the possibility of an evolutionary conserved role of VWF in these structures.

 In order to produce functional VWF activity, high molecular weight multimers are assembled in the trans-Golgi, packaged into WPBs, and secreted. This is followed by circulation in the blood and tethering of platelets to sites of vessel injury, forming the primary platelet plug [6]. It has been previously shown that zebrafish thrombocytes will aggregate in a Vwf-dependent fashion and that morpholino-mediated knockdown results in increased bleeding times and hemorrhage [15]. In this paper we have demonstrated that zebrafish Vwf has the ability to multimerize and form pseudo-WPBs in mammalian cell culture. Taken together, these data suggest that the basic mechanisms of zebrafish Vwf function appear to be conserved.

Previous studies have shown evidence for the presence of the Vwf receptor, GpIb, on thrombocytes in zebrafish and chicken [40, 41]. If thrombocytes bind Vwf as platelets do in mammals, one might expect a high degree of conservation of the Vwf A1 domain, which encodes the GpIb-binding site. The A2 domain, which encodes the Adamts13 cleavage site, is required for the production of properly sized Vwf multimers. When cleavage is reduced, vascular occlusion can occur, while when enhanced, bleeding results [42]. However, there are notable differences between mammalian and non-mammalian vertebrate systems. Despite the overall amino acid similarity and conservation of synteny of Vwf, the A1 and A2 domains display the largest degree of divergence when compared to humans. It is tempting to speculate that the A1 domain has evolved a relatively increased or decreased ability to bind thrombocytes in compensation for the latter's lesser or greater role in the initiation of primary hemostasis. Shear forces required to expose the A1 and A2 domains are likely to be different in zebrafish compared to mammals. Despite their functional similarities, nucleated thrombocytes are clearly different from anucleate platelets, suggesting the possibility that the two function quite differently. Studies of avian thrombocytes, which are also nucleated, have led to the hypothesis that human cardiovascular disease may be related to the existence of platelet rather than thrombocyte-initiated primary hemostasis [41]. Further understanding of the role of thrombocytes and Vwf in zebrafish and avian hemostasis may have potential implications for the treatment of bleeding and thrombotic disorders.

## Figures and Tables

**Table 1:** List of primers and sequences.

Reference number	Sequence	Description
92	AGTCGGCAGCACATACACAC	*vwf *cloning, assembly of fragment 1 (EcoRI-BstBI)
93	ATCCGGACAGGTCAGTTCAC	*vwf *cloning, assembly of fragment 1 (EcoRI-BstBI)
94	CCTGCAGCTTAAACCCAAAG	*vwf *cloning, assembly of fragment 2 (BstBI-AvaI)
95	AAAGCTTCATCGTCCAGCTC	*vwf *cloning, assembly of fragment 2 (BstBI-AvaI)
96	CTGTTGACGGCAAGTGCTAA	*vwf *cloning, assembly of fragment 3 (AvaI-SbfI)
97	TCTCCTGATGCTGGACACAC	*vwf *cloning, assembly of fragment 3 (AvaI-SbfI)
98	GACGGCAGTGTAACGACAGA	*vwf *cloning, assembly of fragment 4 (SbfI-ApaLI)
99	CCTGCAAGAGAGCCGATAAC	*vwf *cloning, assembly of fragment 4 (SbfI-ApaLI)
116	TGCGTGCTGAATCAAACTGT	*vwf *cloning, 3′ RACE (ApaLI-SpeI), SpeI vector derived
128	AGTCGCCAGGGAATTCATAA	*vwf *cloning, 5′ RACE (NotI-EcoRI), NotI vector derived
130	TTTGATTGACATTTTTATTTATTGTAGTTTA	*vwf *cloning, amplification of 3′ UTR
543	gatttaggtgacactatagCGACATGCAAGTGCAGAAGT	424 bp *vwf *riboprobe (exon 28) with SP6 promoter overhang
544	taatacgactcactatagggGCTGGGTTTTGCTGTAGGAG	424 bp *vwf *riboprobe (exon 28) with T7 promoter overhang
545	gatttaggtgacactatagGGAGTTATCGGCTCTCTTGC	441 bp *vwf *riboprobe (exons 47–52) with SP6 promoter overhang
546	taatacgactcactatagggACACAGACTTGCTGCCACAC	441 bp *vwf *riboprobe (exons 47–52) with T7 promoter overhang

	CTCGAGAGAATTCCACCACACTGGACTAGTGGATC	XhoI-PmeI human VWF linker sequence (includes Myc/HIS tag)
	CGAGCTCGGTACCAAGCTTGGGCCCGAACAAAAAC	
	TCATCTCAGAAGAGGATCTGAATAGCGCCGTCGAC	
	CATCATCATCATCATCATTGAGTTTAAAC	

**Table 2:** Human/zebrafish Vwf domain conservation.

Domain	Identities	Positives	Human	zebrafish
	(%)	(%)	length	length
D1	51	70	352	351
D2	64	79	360	359
D′	51	71	90	88
D3	56	69	376	370
A1	36	57	220	233
A2	28	56	193	193
A3	42	58	202	207
D4	39	54	372	382
B1	58	73	35	34
B2	52	64	26	30
B3	67	83	25	25
C1	50	58	116	107
C2	48	63	119	117
CK	42	64	90	91

Total	46	62	2813	2812

Alignment of human and zebrafish amino acid sequences using BLAST (http://blast.ncbi.nlm.nih.gov/ ). Percentage identity represents exact amino acid matches, while positives indicate conserved substitutions. Domain length is in amino acids.

## References

[1] Davidson CJ, Hirt RP, Lal K (2003). Molecular evolution of the vertebrate blood coagulation network. *Thrombosis and Haemostasis*.

[2] Davidson CJ, Tuddenham EG, McVey JH (2003). 450 million years of hemostasis. *Journal of Thrombosis and Haemostasis*.

[3] Jiang Y, Doolittle RF (2003). The evolution of vertebrate blood coagulation as viewed from a comparison of puffer fish and sea squirt genomes. *Proceedings of the National Academy of Sciences of the United States of America*.

[4] Nichols WC, Ginsburg D (1997). Von Willebrand disease. *Medicine*.

[5] Schneppenheim R, Budde U (2011). Von Willebrand factor: the complex molecular genetics of a multidomain and multifunctional protein. *Journal of Thrombosis and Haemostasis*.

[6] Sadler JE (2009). Von Willebrand factor assembly and secretion. *Journal of Thrombosis and Haemostasis*.

[7] Ginsburg D, Bowie EJW (1992). Molecular genetics of von Willebrand disease. *Blood*.

[8] Levy G, Ginsburg D (2001). Getting at the variable expressivity of von Willebrand disease. *Thrombosis and Haemostasis*.

[9] Jagadeeswaran P, Paris R, Rao P (2006). Laser-induced thrombosis in zebrafish larvae: a novel genetic screening method for thrombosis. *Methods in Molecular Medicine*.

[10] Kidd KR, Weinstein BM (2003). Fishing for novel angiogenic therapies. *British Journal of Pharmacology*.

[11] Chen JN, Haffter P, Odenthal J (1996). Mutations affecting the cardiovascular system and other internal organs in zebrafish. *Development*.

[12] Stainier DY, Fouquet B, Chen JN (1996). Mutations affecting the formation and function of the cardiovascular system in the zebrafish embryo. *Development*.

[13] Jin SW, Herzog W, Santoro MM (2007). A transgene-assisted genetic screen identifies essential regulators of vascular development in vertebrate embryos. *Developmental Biology*.

[14] Patton EE, Zon LI (2001). The art and design of genetic screens: zebrafish. *Nature Reviews Genetics*.

[15] Carrillo M, Kim S, Rajpurohit SK, Kulkarni V, Jagadeeswaran P (2010). Zebrafish von Willebrand factor. *Blood Cells, Molecules, and Diseases*.

[16] Dang LT, Purvis AR, Huang RH, Westfield LA, Sadler JE (2011). Phylogenetic and functional analysis of histidine residues essential for pH-dependent multimerization of von willebrand factor. *Journal of Biological Chemistry*.

[17] Fujita PA, Rhead B, Zweig AS (2011). The UCSC genome browser database: update 2011. *Nucleic Acids Research*.

[18] Goujon M, McWilliam H, Li W (2010). A new bioinformatics analysis tools framework at EMBL-EBI. *Nucleic Acids Research*.

[19] Larkin MA, Blackshields G, Brown NP (2007). Clustal W and clustal X version 2.0. *Bioinformatics*.

[20] Buchner DA, Su F, Yamaoka JS (2007). Pak2a mutations cause cerebral hemorrhage in redhead zebrafish. *Proceedings of the National Academy of Sciences of the United States of America*.

[21] Haberichter SL, Fahs SA, Montgomery RR (2000). Von Willebrand factor storage and multimerization: 2 independent intracellular processes. *Blood*.

[22] Schullek J, Jordan J, Montgomery RR (1984). Interaction of von Willebrand factor with human platelets in the plasma milieu. *Journal of Clinical Investigation*.

[23] Westerfield M (2000). *The Zebrafish Book. A Guide For the Laboratory Use of Zebrafish (Danio Rerio)*.

[24] Thisse C, Thisse B (2008). High-resolution in situ hybridization to whole-mount zebrafish embryos. *Nature Protocols*.

[25] Sullivan-Brown J, Bisher ME, Burdine RD (2011). Embedding, serial sectioning and staining of zebrafish embryos using JB-4 resin. *Nature Protocols*.

[26] Rehemtulla A, Kaufman RJ (1992). Preferred sequence requirements for cleavage of pro-von Willebrand factor by propeptide-processing enzymes. *Blood*.

[27] Xiang Y, de Groot R, Crawley JTB, Lane DA (2011). Mechanism of von Willebrand factor scissile bond cleavage by a disintegrin and metalloproteinase with a thrombospondin type 1 motif, member 13 (ADAMTS13). *Proceedings of the National Academy of Sciences of the United States of America*.

[28] Michaux G, Hewlett LJ, Messenger SL (2003). Analysis of intracellular storage and regulated secretion of 3 von Willebrand disease-causing variants of von Willebrand factor. *Blood*.

[29] Wang JW, Valentijn KM, de Boer HC (2011). Intracellular storage and regulated secretion of von Willebrand factor in quantitative von Willebrand disease. *Journal of Biological Chemistry*.

[30] Ginsburg D, Handin RI, Bonthron DT (1985). Human von Willebrand Factor (vWF): isolation of complementary DNA (cDNA) clones and chromosomal localization. *Science*.

[31] Lynch DC, Zimmerman TS, Collins CJ (1985). Molecular cloning of cDNA for human von Willebrand factor: authentication by a new method. *Cell*.

[32] Sadler JE, Shelton-Inloes BB, Sorace JM (1985). Cloning and characterization of two cDNAs coding for human von Willebrand factor. *Proceedings of the National Academy of Sciences of the United States of America*.

[33] Verweij CL, de Vries CJM, Distel B (1985). Construction of cDNA coding for human von willebrand factor using antibody probes for colony-screening and mapping of the chromosomal gene. *Nucleic Acids Research*.

[34] ISTH SSC VWF Database http://www.vwf.group.shef.ac.uk/index.html.

[35] Ekker SC, Stemple DL, Clark M, Chien CB, Rasooly RS, Javois LC (2007). Zebrafish genome project: bringing new biology to the vertebrate genome field. *Zebrafish*.

[36] Yamamoto K, de Waard V, Fearns C, Loskutoff DJ (1998). Tissue distribution and regulation of murine von Willebrand factor gene expression in vivo. *Blood*.

[37] Wick MR, Hornick JL, Dabbs DJ (2010). Immunohistology of soft tissue and osseous neoplasms. *Diagnostic Immunohistochemistry*.

[38] Schier AF (2007). The maternal-zygotic transition: death and birth of RNAs. *Science*.

[39] Kimmel CB, Ballard WW, Kimmel SR, Ullmann B, Schilling TF (1995). Stages of embryonic development of the zebrafish. *Developmental Dynamics*.

[40] Jagadeeswaran P, Sheehan JP, Craig FE, Troyer D (1999). Identification and characterization of zebrafish thrombocytes. *British Journal of Haematology*.

[41] Schmaier AA, Stalker TJ, Runge JJ (2011). Occlusive thrombi arise in mammals but not birds in response to arterial injury: evolutionary insight into human cardiovascular disease. *Blood*.

[42] Sadler JE (2005). Von Willebrand factor: two sides of a coin. *Journal of Thrombosis and Haemostasis*.

