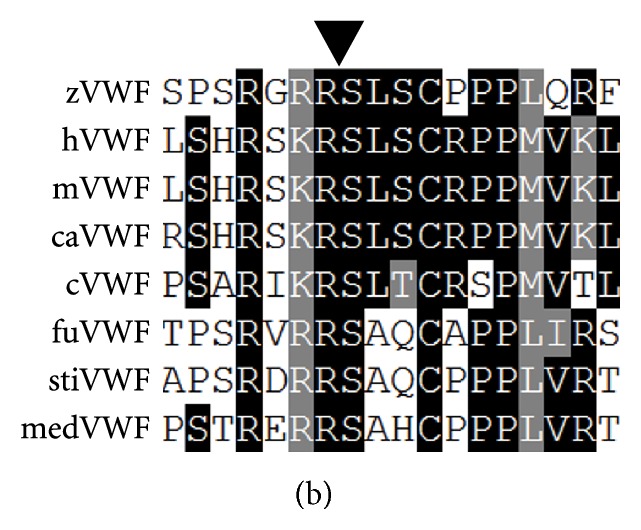# Corrigendum to “Characterization of Zebrafish von Willebrand Factor Reveals Conservation of Domain Structure, Multimerization, and Intracellular Storage”

**DOI:** 10.1155/2015/526854

**Published:** 2015-08-19

**Authors:** Arunima Ghosh, Andy Vo, Beverly K. Twiss, Colin A. Kretz, Mary A. Jozwiak, Robert R. Montgomery, Jordan A. Shavit

**Affiliations:** ^1^Life Sciences Institute, University of Michigan, Ann Arbor, MI 48109, USA; ^2^Department of Pediatrics, University of Michigan, Room 8301 Medical Science Research Building III, 1150 W. Medical Center Drive, Ann Arbor, MI 48109-5646, USA; ^3^Blood Research Institute, Medical College of Wisconsin, Milwaukee, WI 53226, USA

In the paper by Ghosh et al. titled “Characterization of Zebrafish von Willebrand Factor Reveals Conservation of Domain Structure, Multimerization, and Intracellular Storage” [1] a correction should be noted. It was originally stated that “The propeptide cleavage site, Arg-Ser, is highly conserved across all species examined except for medaka, and is a part of the extended RX(R/K)R motif (Figure 1(b))…. There was one cysteine present solely in medaka, four residues N-terminal to the propeptide cleavage site, but its absence in other species makes its significance unclear.” After further review, we note that the medaka sequence was from an older draft of the genome. Examination of the most recent medaka sequence shows conservation of the extended RX(R/K)R motif and that there is not a cysteine present four residues N-terminal to the propeptide cleavage site. A corrected Figure 1(b) is provided below. Other than the statements above, this does not alter the conclusions or interpretations presented in the paper. The authors would like to acknowledge Evan Sadler (Washington University) for calling their attention to this discrepancy.

## Figures and Tables

**Figure 1:**